# Subtype specific expression and survival prediction of pivotal lncRNAs in muscle invasive bladder cancer

**DOI:** 10.1038/s41598-020-77252-2

**Published:** 2020-11-24

**Authors:** Sebastien Rinaldetti, Thomas Stefan Worst, Eugen Rempel, Maximilian C. Kriegmair, Arndt Hartmann, Stefan Porubsky, Christian Bolenz, Philipp Erben

**Affiliations:** 1 Department of Hematology and Oncology, University Medical Centre Mannheim, Theodor-Kutzer-Ufer 1-3, 68167 Mannheim, Germany; 2 Department of Hematology and Oncology, Centre Hospitalier de Luxembourg, Luxembourg, Luxembourg; 3 Division of Signalling and Functional Genomics, German Cancer Research Center (DKFZ), Im Neuenheimer Feld 280, 69120 Heidelberg, Germany; 4 Department of Urology and Urosurgery, Medical Faculty Mannheim, University of Heidelberg, Theodor-Kutzer-Ufer 1-3, 68167 Mannheim, Germany; 5 Institute of Pathology, Heidelberg University Hospital, Im Neuenheimer Feld 230, 69120 Heidelberg, Germany; 6 Institute of Pathology, University of Erlangen-Nuremberg, Krankenhausstraße 8-10, 91054 Erlangen, Germany; 7 Institute of Pathology, University Medical Center Mainz, Langenbeckstraße 1, 55131 Mainz, Germany; 8 Department of Urology, University of Ulm, Prittwitzstraße 43, 89075 Ulm, Germany

**Keywords:** Cancer, Urological cancer

## Abstract

Comprehensive transcriptome expression analyses of bladder cancer revealed distinct lncRNA clusters with differential molecular and clinical characteristics. In this study, pivotal lncRNAs were assessed for their impact on survival and their differential expression between the molecular bladder cancer subtypes. FFPE samples from chemotherapy-naïve patients with muscle invasive bladder cancer (MIBC) were analyzed on the Nanostring nCounter platform for absolute quantification. An established 36-gene panel was used for molecular subtype classification into basal, luminal and infiltrated MIBC. In a second step, 14 pivotal lncRNAs were assessed for their molecular subtype attribution, and their predictive value in disease-specific survival. In silico validation was performed on a total of 487 MIBC patients (MDA, TGCA and Chungbuk cohort). Several pivotal lncRNAs showed a distinct molecular subtype attribution: e.g. MALAT1 showed a downregulation in the basal subtype (*p* = 0.009), TUG1 and CBR3AS1 showed an upregulation in the luminal subtype (*p* ≤ 0.001). High transcript levels of SNHG16, CBR3AS1 and H19 appeared to be predictive for a shorter disease-specific survival. Patients overexpressing putative oncogenes MALAT1 and TUG1 in MIBC tissue presented prolonged survival, suggesting tumor suppressive effects of both lncRNAs. The Nanostring nCounter proved to be a valid platform for the quantification of low-abundance transcripts including lncRNAs.

## Introduction

The estimated number of bladder cancer related deaths in the US is comprised of 61,000 male and 18,770 female cases, according to the 2019 cancer data from the American Cancer Society^1^. Indeed, bladder cancer is in the top ten cancer-related causes of death in men. Recent findings revealed a pronounced molecular heterogeneity in muscle invasive (MIBC) and non-muscle invasive bladder cancer (NMIBC)^2–4^. Similar to breast cancer, bladder cancer can be classified into heterogeneous molecular subtypes which are related to treatment response and survival^2,5,6^. These subtypes are based on transcriptome data, and cluster MIBC patients in up to 5 or 6 different molecular subtypes, with strongest evidence for basal, luminal and mesenchymal/infiltrated subtypes^7^. The TCGA working group showed that similar MIBC subtypes can also be identified when only considering long non-coding RNA (lncRNA) expression data^2^. This is of particular interest since lncRNA expression is known to be more cellular phenotype- or tissue-specific when compared with mRNA^8^. LncRNAs are non-protein-coding RNA molecules greater than 200 nucleotides in length which serve as central regulators of gene expression. Little is known about their functionality given the lack of sequence conservation across species and the lack of functional studies. Furthermore, the overall expression of lncRNAs is tenfold lower than mRNA transcripts, which makes their quantification challenging when using high-throughput methods based on relative quantification (e.g. transcriptome microarrays)^9^. Due to their relative low abundance many lncRNAs may also be lost during data processing. Thus, only a few lncRNAs will remain eligible for further investigation (Table 1). Hence this study aimed to investigate the expression of pivotal lncRNAs known to have functional relevance in genitourinary cancers, on the sensitive Nanostring nCounter platform for absolute RNA quantification^10,11^. We focused on 14 lncRNAs known to be either oncogenic (CTBP1AS, MALAT1, UCA1), tumor-suppressive (GAS5, MEG3, TP53COR1), and with prognostic (H19, SNHG16, TUG1, XIST, HOTAIR BLACAT1) or therapeutic relevance (CBR3AS1, SRA1)^12–28^. By investigating these lncRNAs in an in-house MIBC patient cohort and by validating and complementing our data by in silico analyses of three publicly available cohorts, we aimed to gain further insight into the clinical impact of these lncRNAs and their differential expression between the different MIBC molecular subtypes.

**Table 1:**
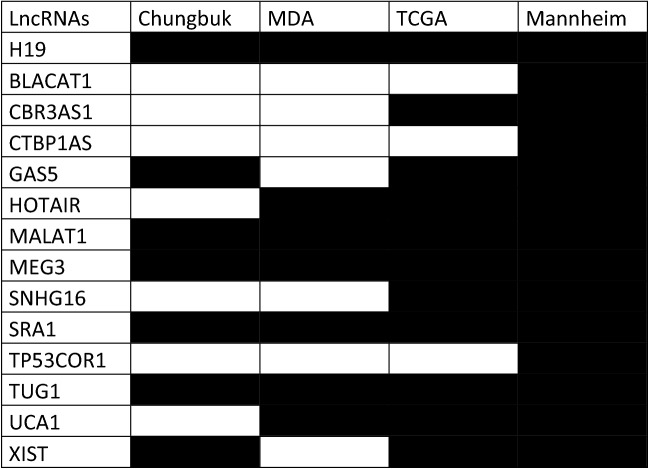
Summary of available expression data of 14 pivotal lncRNA relevant in genitourinary cancers.

Black = Expression data available.

## Results

### Clinicopathologic characteristics and subtype clusters

**Figure 1:**
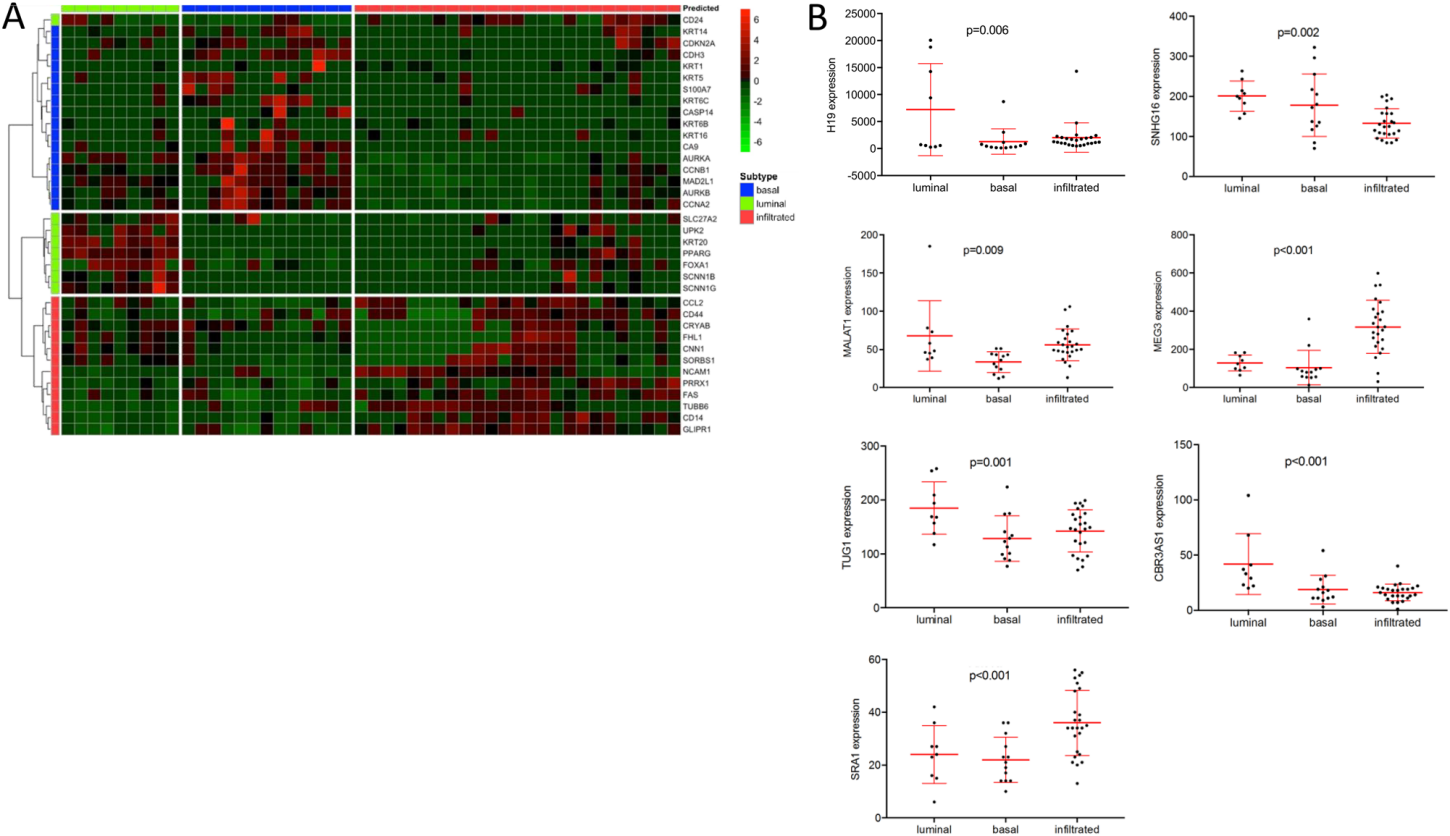
(**A**) Heatmap of the Mannheim cohort (n = 47) clustering patients into the basal, luminal and infiltrated subtypes based on the in-house 36-consensus gene panel (**B**) LncRNAs with significant differential expression between the molecular MIBC subtypes based on the Mannheim cohort.

Clinicopathologic characteristics of the Mannheim cohort (n = 47) are summarized in Table 2. Clinicopathologic characteristics of the Chungbuk (n = 61), MDA (n = 55) and TCGA (n = 371) cohorts have been described before^2,29,30^. A Nanostring nCounter-based consensus gene panel of 36 protein-coding genes based on previous work of our group (30) allowed a consistent clustering of tumor samples throughout the cohorts, classifying MIBC into basal, luminal and infiltrated subtype (Fig. 1A). This molecular taxonomy is based on the MDA subtypes, however given the enrichment of immune gene signatures in the p53-like subtype, the designation of ‘infiltrated’ seemed more accurate for this group of tumors^31^. As the lncRNA panel was not included in the 36-gene set used for subtype clustering, this study allows a clustering-independent assessment of the lncRNAs of interest.

**Table 2:** Clinicopathologic characteristics of the Mannheim cohort.

Cohort characteristics	Total	(%)
Cohort size	47	
Median age	67	
Female	13	(28)
Male	34	(72)
**TNM stage**		
pTa*, pT1*, pTis*	3	(6)
pT2	11	(23)
pT3	26	(55)
pT4	7	(15)
pN +	17	(36)
cM +	8	(17)
**Additional therapy**		
NAC	1	(2)
AC	7	(16)

*AC* adjuvant chemotherapy, *NAC* neoadjuvant chemotherapy.

*Muscle-invasive at TUR.

### Subtype-specific lncRNA expression

The Nanostring nCounter proved to be a valid method for the detection and absolute quantification of lncRNAs, as all 14 lncRNAs could be detected and quantified in our in-house cohort. Based on the subtypes identified using the consensus cluster genes described above, the overall distribution of lncRNA transcripts was assessed between subtypes. Seven of the 14 lncRNAs showed a significant differential expression between subtypes in the MIBC Mannheim cohort (Fig. 1B). TUG1 and CBR3AS1 were overexpressed in the luminal subtype (*p* ≤ 0.001, Fig. 1B). A specific upregulation in the infiltrated subtype was found for SRA1 and MEG3 (*p* < 0.001, Fig. 1B). Expression of MALAT1 and H19 was reduced in the basal subtype (*p* < 0.01), whereas SNHG16 was downregulated in the infiltrated subtype (*p* = 0.002, Fig. 1B).

**Figure 2:**
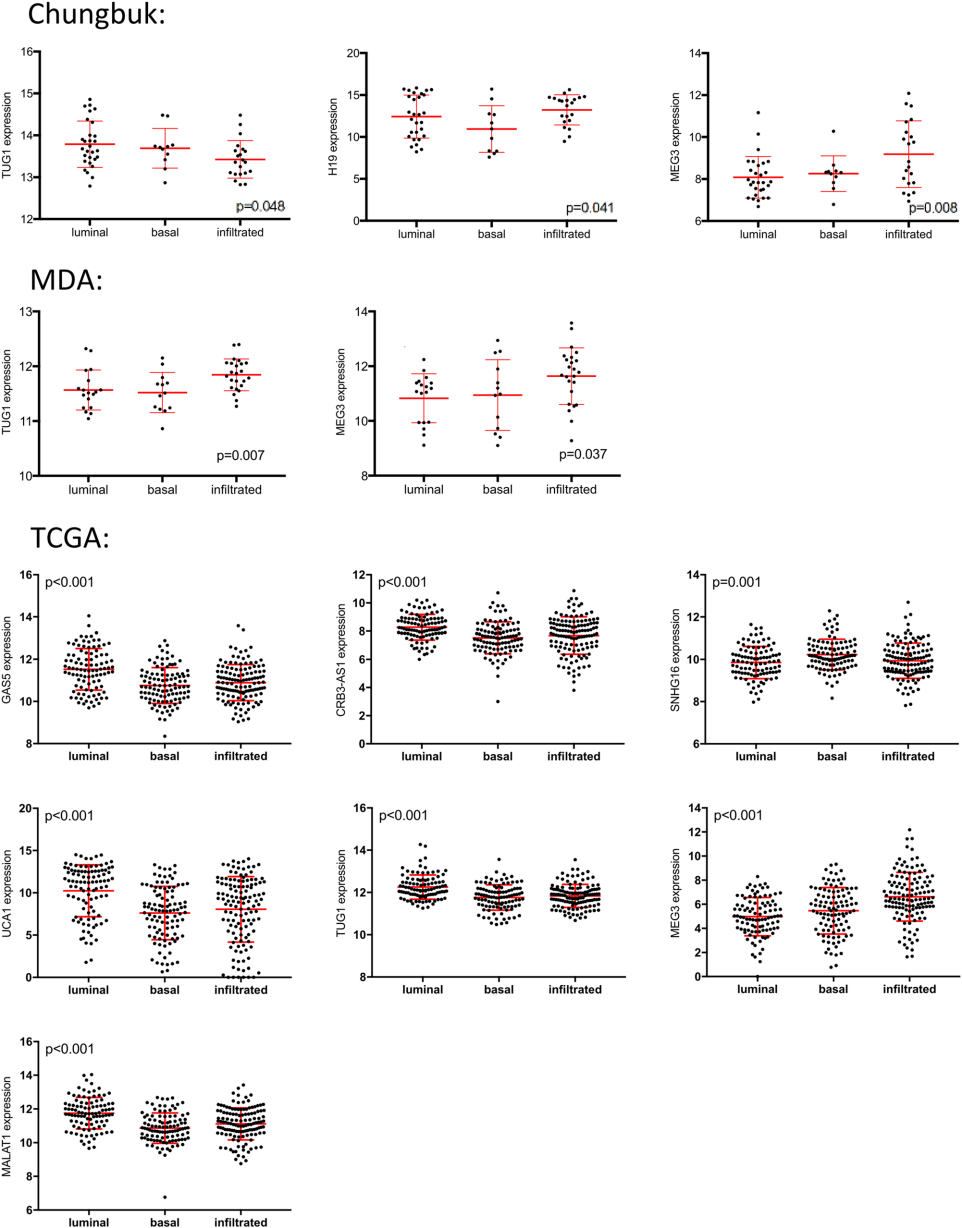
Differential lncRNA expression between the molecular MIBC subtypes based on the Chungbuk (n = 61), MDA (n = 55) and TCGA (n = 371) cohorts. The patients were clustered choosing the same 36-consensus genes than in the Mannheim cohort, as described before^31^.

We further investigated the microarray-based MDA (GSE48276) and Chungbuk (GSE16507) datasets as well as the RNA-seq-based TCGA (cBioportal) transcriptome data, by means of a cross-platform validation^2,29,30^. From the 14 lncRNAs investigated in this study, only few are covered through the whole transcriptome analysis platforms (Chungbuk: 7/14, MDA: 7/14, TCGA: 11/14) (Table 1). In all three validation cohorts MEG3 showed an exclusive subtype specific expression in the infiltrated subtype (*p* < 0.05, Fig. 2). For H19, the Chungbuk cohort confirmed its basal suppression (*p* = 0.041), whereas the MDA and TCGA cohort indicated the same trend but not statistically significant. The overexpression of SRA1 in the infiltrated subtype could not be confirmed in the validation data sets (Fig. 2).

Similar to the Mannheim cohort, the Chungbuk and TCGA cohorts showed an upregulation of TUG1 in the luminal subtype (*p* = 0.048 and *p* < 0.001 respectively, Fig. 2). This trend was not confirmed in the MDA cohort. MALAT1 showed lower transcript levels in the basal and infiltrated subtypes of the TCGA cohort (*p* < 0.001, Fig. 2), confirming the findings in the Mannheim cohort. However, MALAT1 was not significantly altered in the Chungbuk and MDA cohorts. In silico data for CBR3AS1 and SNGH16 were only available in the TCGA cohort (Table 1). Similar to the Mannheim cohort, the TCGA cohort showed an enrichment of CBR3AS1 in the luminal subtype (*p* < 0.001, Fig. 2). In the TCGA cohort, SNGH16 was overexpressed in the basal subtype (*p* = 0.001) but showed no downregulation in the infiltrated subtype as in the Mannheim cohort. Only the TCGA cohort showed a subtype specific expression for GAS5 by its high transcript levels in the luminal subtype (*p* < 0.001, Fig. 2).

When compared to the TCGA-based subtypes (basal squamous, luminal, luminal infiltrated, luminal papillary, neuronal), the lncRNAs showed a similar subtype specific differential expression than with the consensus gene set described above (luminal, basal and infiltrated) (Figure S1). Subtype specific expression of BLACAT1, CTBP1AS, UCA1, HOTAIR, XIST and TP53COR1 was not seen in any of the cohorts.

### MIBC survival prediction by lncRNAs

**Figure 3:**
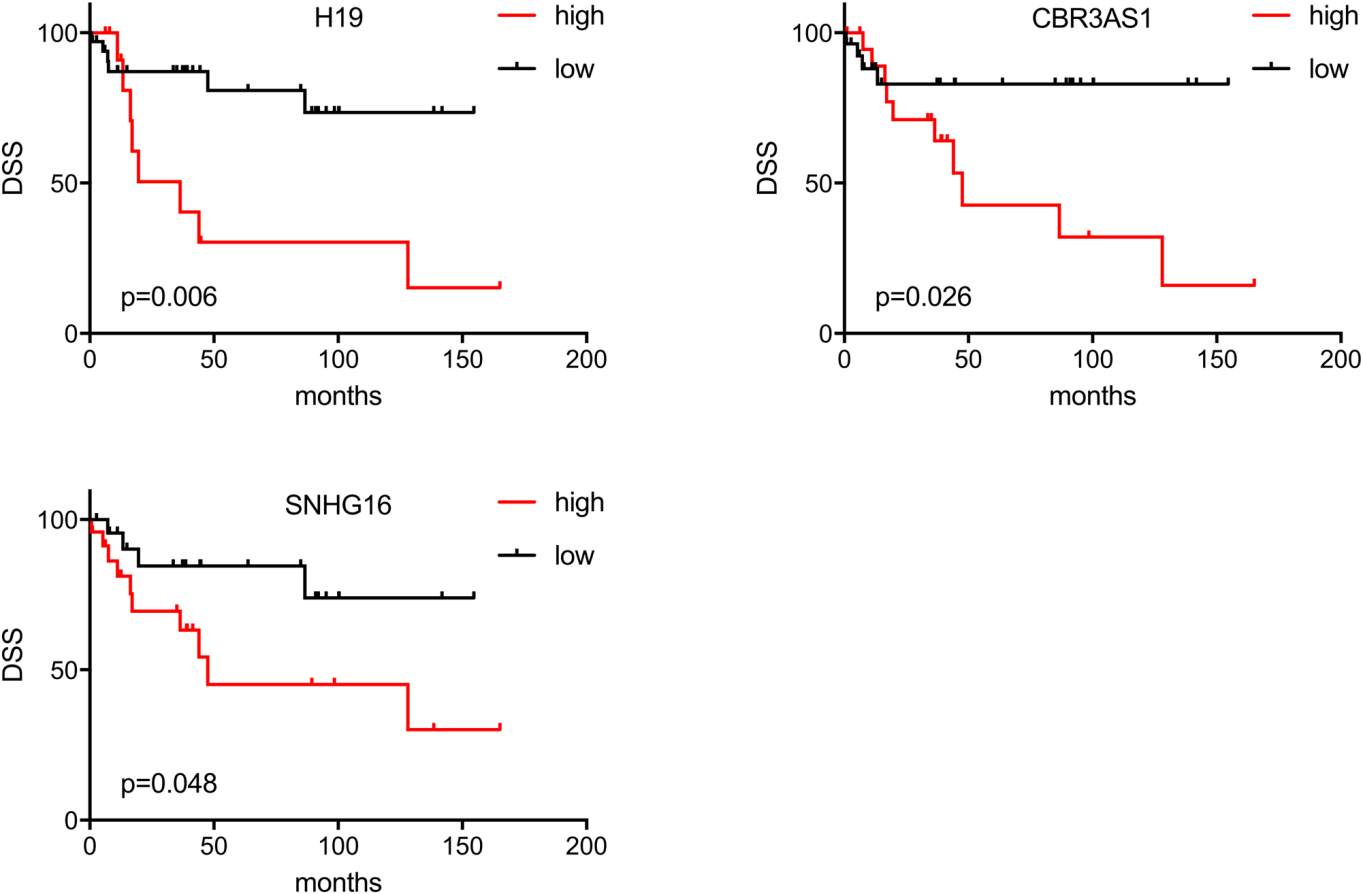
Kaplan Meier plots of lncRNAs allowing a significant risk stratification (*p* < 0.05) based on high and low transcript level expression in the Mannheim cohort. *DSS* disease specific survival.

For the Mannheim cohort, we assessed disease-specific survival (DSS) in 47 chemotherapy-naïve MIBC patients in order to investigate the correlation of differentially expressed lncRNAs with the natural disease course. Expression levels of H19, CBR3AS1 and SNHG16 allowed a distinct risk stratification. Overexpression of all three lncRNA genes was associated with DSS (*p* < 0.05) in MIBC. Higher expression of CBR3AS1, H19 and SNGH16 was associated with adverse outcome in MIBC for DSS (*p* = 0.026, *p* = 0.006 and *p* = 0.048 respectively, Fig. 3). CBR3AS1 and SNHG16 showed also significance in OS (*p* = 0.05 and *p* = 0.016 respectively, Figure S2). In the multiple Cox regression analysis H19 (HR 5.12, CI 95% 1.6–16.4, *p* = 0.006) and CBR3AS1 (HR 4.4, CI 95% 1.2–16.1, *p* = 0.026) emerged as independent predictors for DSS, whereas SNGH16 was not retained in the regression model.

### Validation of survival data by the TCGA, MDA and Chungbuk cohort

**Figure 4:**
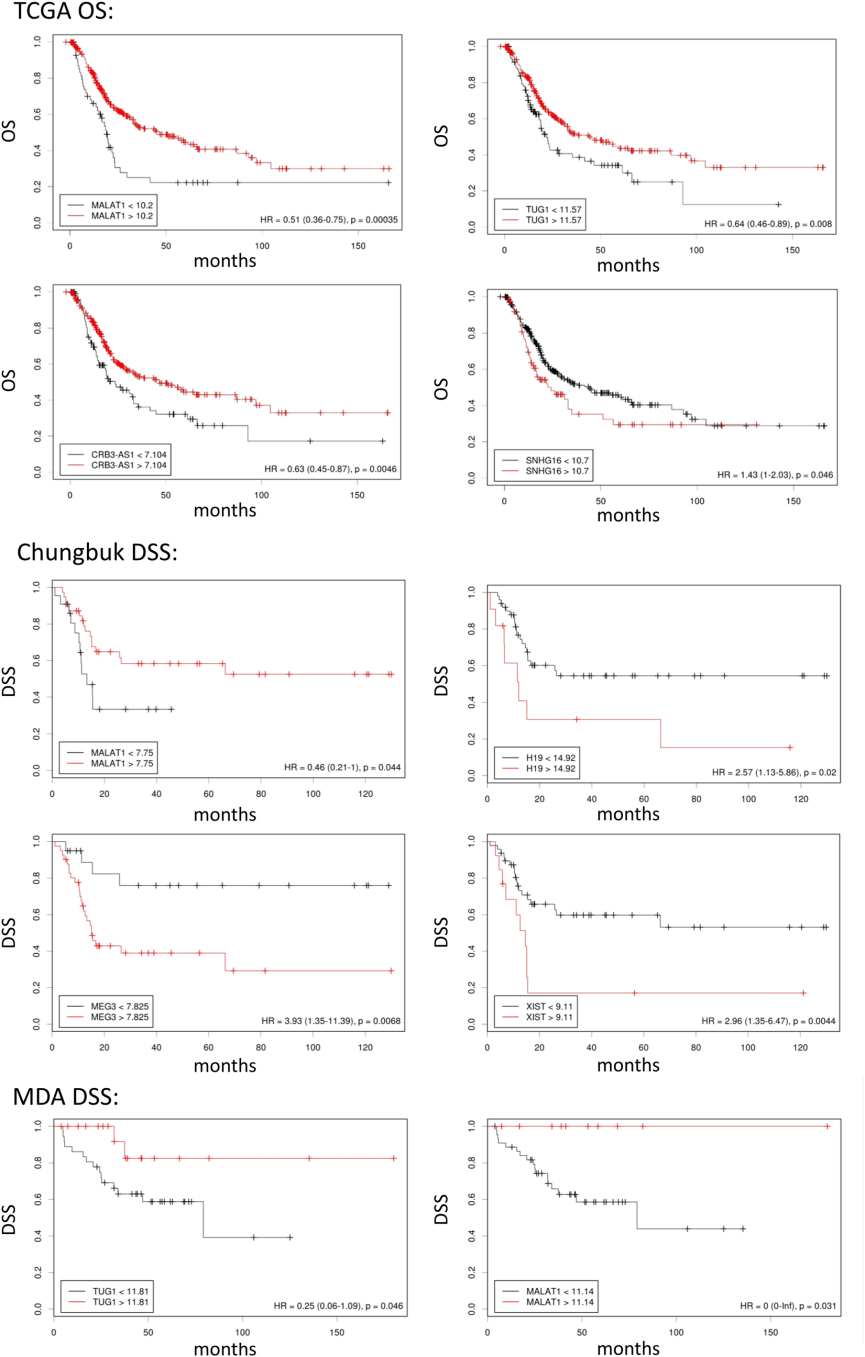
Kaplan Meier plots of lncRNAs allowing a significant risk stratification (*p* < 0.05) based on high and low transcript level expression. In silico validation of lncRNA based survival prediction on behalf of the TCGA (n = 371, no DSS data available), Chungbuk (n = 61) and MDA (n = 55) cohort. Only genes with a valid cutoff are shown. *OS* overall survival, *DSS* Disease specific survival.

The hazard ratios of all lncRNAs analyzed in this study are summarized in table S1. Patients with higher CBR3AS1 in the TCGA cohort, showed increased OS (Fig. 4). Furthermore, CBR3AS1 was overexpressed in the luminal and luminal papillary subtypes of the TCGA cohort, which are known for their good prognosis (Fig. 2 and Figure S1). The Chungbuk cohort confirmed a lower DSS for MIBC with high H19 expression (Fig. 4, *p* = 0.02). However, the other cohorts didn’t confirm this finding. This might be explained by the differences in MIBC therapy between the cohorts together with the fact that only OS and no DSS data are available for the TCGA cohort.

The MDA and the TCGA cohorts showed a better outcome for patients overexpressing TUG1 (Fig. 4). XIST is known for promoting cancer proliferation and migration, however only the Chungbuk cohort showed a correlation with unfavorable DSS (Fig. 4, *p* = 0.0044). Also, for MEG3 the predictive power seemed weak, as only in the Chungbuk cohort an association between high expression and a poor prognosis was seen (*p* = 0.0068). However, it is of note that several in vitro studies suggested this gene to be a tumor suppressor, thus further clinical validation is needed.

Interestingly, MALAT1 showed a survival advantage in all in silico cohorts when highly expressed (Fig. 4), and stratification in TCGA subtypes showed overexpression of MALAT1 in the luminal papillary subtype, which is known for its particularly favorable overall survival (Figure S1)^2^.

## Discussion

This study took a closer look at known hallmark oncogenic, tumor suppressive and predictive lncRNAs in the context of bladder cancer subtypes and outcome prediction. Based on a focused Nanostring nCounter-based consensus gene panel, our in-house cohort of chemotherapy-naive patients with urothelial carcinoma was clustered into a basal, a luminal and an infiltrated subtype. This absolute quantification assay showed a good sensitivity for the subtype attribution of lncRNAs in a clustering-independent manner. The MDA and Chungbuk cohort data are both based on microarray analysis whereas TCGA gene expression was determined by RNA-seq. All cohorts included T2-T4 tumors. Only the MDA cohort included up to 23% of patients with neoadjuvant treatment and no adjuvant treatment. In contrast, 43% of the patients in the Chungbuk cohort received adjuvant Cisplatin-based therapy, no patient received neoadjuvant treatment. The MDA cohort further included 22% of samples derived from TURBT, whereas the samples from all other studies were exclusively obtained by cystectomy. Thus, a weakness of the biomarker in silico validation, for bladder cancer in general, is the clinical heterogeneity of public available cohorts. Nonetheless, for most lncRNAs analyzed in this study, the subtype-specific expression could be validated and reproduced by in silico analyses from the independent TCGA, MDA and Chungbuk cohorts.

Here, we showed for the first time that high expression of CBR3AS1 and SNGH16 contributes to adverse outcome in MIBC for DSS and OS. Both genes are known to promote tumor growth in vitro, in bladder or prostate cancer^10^. Only CBR3AS1 showed a major survival discrepancy between the Mannheim and TCGA cohort. However, Cox regression analysis showed that CBR3AS1 as well as H19 are independent DSS predictors. CBR3AS1 is known to have a negative impact on survival and to promote epithelial-mesenchymal transitions and proliferation in several cancer entities. Thus, further validation is needed with a sensitive quantification method on a larger cohort, especially since this lncRNA is a candidate therapeutic target and the TCGA cohort had no DSS data available at the time of this study^12,32^.

TUG1 is known to be upregulated in the basal subtypes of breast cancer and bladder cancer as well as in squamous tissue in various cancer types^33,34^. However, our study showed a downregulation in the basal subtype of the Mannheim and TCGA cohorts. This is because all squamous carcinomas were removed from both cohorts for the sake of reproducibility of molecular and clinical data (Figure S3). According to similar clustering approaches (e.g. MDA and TCGA) samples with squamous histology are known to cluster to the basal subtype. Despite their different biology and highly negative impact on survival, those samples were rarely excluded from studies, giving rise to contradictory results. In the present study, this resulted in an enrichment of MALAT1 and TUG1 in the luminal papillary TCGA subtype and infiltrated subtype (Mannheim data), which have shown to have a favorable outcome^2,31^. Interestingly, despite their proposed oncogenic functions, we demonstrated that MALAT1 and TUG1 represent predictors of favorable outcome in muscle-invasive urothelial carcinoma. A recent preclinical in vivo study identified MALAT1 as a potential tumor suppressor, preventing the formation of metastases in breast cancer^35^. Concerning TUG1, the findings of a recent study are in accordance with our data, validating its role as a biomarker for favorable outcome^33^. As the functionality of TUG1 in bladder cancer is still unknown, further studies are needed with regard to a possible tumor suppressive effect.

To date very little is known about the prognostic value and function of SNGH16 in bladder cancer. However, the TCGA MIBC study was able to cluster patients into 4 lncRNA groups. SNHG16 showed a distinct overexpression in the lncRNA cluster 2, characterized by the highest risk of death of all the four lncRNA clusters. In both the Mannheim and TCGA cohorts, SNGH16 upregulation correlated with higher risk of DSS. However, SNGH16 was not an independent predictor of DSS, after multiple regression analysis.

Gene expression studies of many pivotal lncRNAs are still sparse. In fact, these genes tend to be excluded during the processing of transcriptomic data, due to their low abundance (Table 1). Sequencing depth and the sensitivity of the quantification method are crucial for the elucidation of the role of lncRNAs in translational studies. Given the overlapping differential expression of lncRNAs throughout the cohorts, they undoubtedly also play a role in subtype/phenotype determination or differentiation. Yet, the underlying biological mechanisms still need to be elucidated. To date no specific in vitro studies have been described in the literature for the great majority of lncRNAs. However, it is known that lncRNA expression patterns are highly tumor- and tissue-specific. The integration of lncRNAs in subtyping gene sets, might help to reduce the total number of genes needed for an accurate and standardized subtype attribution of patient samples. In this study, the impact of several lncRNAs on MIBC survival has been demonstrated. The lack of biological or functional understanding of those genes urge further in vitro and in vivo studies given their potential translational benefit in diagnostics and clinics.

## Materials and methods

### MIBC patient cohorts

The Mannheim cohort represents a collection of 47 formalin-fixed paraffin-embedded (FFPE) samples of muscle-invasive urothelial carcinoma tissue. Most samples from the University Medical Center Mannheim were collected from chemotherapy-naïve patients during radical cystectomy with bilateral lymphadenectomy, only 2 patients received neoadjuvant chemotherapy and 7 patients received adjuvant chemotherapy. All the study procedures were carried out in accordance to the declaration of Helsinki. The FFPE specimens were reviewed by a uropathologist (AH) according to the TNM classification of 2010 (UICC). All patients included in this study gave written informed consent and the study was approved by our institutional review board (Ethics commission II of the Heidelberg University, ID: #2013-517N-MA and #2016-814R-MA).

In silico transcriptome expression data of the MDA (n = 55, GSE48276), Chungbuk (n = 61, GSE13507) and TCGA (n = 371, cBioportal) cohorts were used for data validation^2,29,30^. Squamous cell carcinoma cases were excluded, given their different biological and clinical characteristics.

### Gene expression profiling

Nanostring nCounter-based absolute quantification of 36 mRNA transcripts and 14 lncRNAs was performed on FFPE MIBC samples. All samples included a minimum amount of 30% tumor tissue, based on pathological evaluation of matched hematoxylin and eosin stained sections. RNA isolation of 10 µm FFPE sections was performed by a bead-based system (XTRACT kit, STRATIFYER Molecular Pathology GmbH, Cologne, Germany) and the RNA quality was assessed by Nanodrop and qRT-PCR.

Expression data was preprocessed with nSolver software v2.5. Data was normalized using the geometric mean of 6 housekeeping genes (CALM2, RPL37A, B2M, TUBB, GAPDH and G6PD) together with 6 internal positive controls. Negative internal controls (n = 8) were used for negative background subtraction.

The validation datasets were downloaded from cBioportal and the Gene Expression Omnibus. The raw intensities were log2 transformed and quantile normalized. The patients were clustered into three groups by unsupervised hierarchical clustering with Pearson’s correlation as a measure of similarity and Ward as agglomeration method, based on a 36-consensus gene panel as described previously^31^.

### Statistical methods

Clinicopathologic characteristics and differential expression were compared using one-way ANOVA. Overall survival (OS) and disease-specific survival (DSS) were analyzed by the Kaplan–Meier method and tested for significance by the log-rank test. Cutoffs were determined by the minimum *p*-value approach^36^. Only cutoffs that dichotomize the cohort in groups including > 10% of the patients were retained. Univariate and multivariate analyses were performed by a Cox proportional hazards regression model with a forward selection method. Values included in the regression model had statistical significance (*p* < 0.05) in univariate analysis: T2 vs. T3–T4, N + vs. N0, AC vs. no AC. The study has an exploratory character. Statistics were conducted using R version 3.3.1., Graph Pad Prism v7, SAS JMP14 and SPSS v20.0.

## Supplementary information

Supplementary figures.

Supplementary Table S1.
