# Supplementary material for: Subtype specific expression and survival prediction of pivotal lncRNAs in muscle invasive bladder cancer
Source: Sci Rep. 2020 Nov 24;10:20472. doi: 10.1038/s41598-020-77252-2 (PMC7687888; doi:10.1038/s41598-020-77252-2)
Supplement: Supplementary file 1 — Supplementary figures. [file 41598_2020_77252_MOESM1_ESM.pdf]

Subtype specific expression and survival prediction of pivotal lncRNAs in muscle invasive bladder cancer

Sebastien Rinaldetti[1,2§‡], Thomas Stefan Worst[3,4§‡], Eugen Rempel[5], Maximilian C. Kriegmair[4‡], Arndt Hartmann[6‡], Stefan Porubsky[7‡], Christian Bolenz[8‡], Philipp Erben[4‡*]

1. Department of Hematology and Oncology, University Medical Centre Mannheim, Theodor-Kutzer-Ufer 1-3, 68167 Mannheim, Germany
2. Department of Hematology and Oncology, Centre Hospitalier de Luxembourg, Luxembourg
3. German Cancer Research Center (DKFZ), Division of Signalling and Functional Genomics, Im Neuenheimer Feld 280, 69120 Heidelberg, Germany
4. Department of Urology and Urosurgery, University Medical Centre Mannheim, Theodor-Kutzer-Ufer 1-3, 68167 Mannheim, Germany
5. Institute of Pathology, Heidelberg University Hospital, Im Neuenheimer Feld 230, 69120 Heidelberg, Germany
6. Institute of Pathology, University of Erlangen-Nuremberg, Krankenhausstraße 8-10, 91054 Erlangen, Germany
7. Institute of Pathology, University Medical Center Mainz, Langenbeckstraße 1, 55131 Mainz, Germany
8. Department of Urology, University of Ulm, Prittwitzstraße 43, 89075 Ulm, Germany

§equal contribution
‡On behalf of the BRIDGE Consortium
*Corresponding author

Figure S1: TCGA Subtypes:

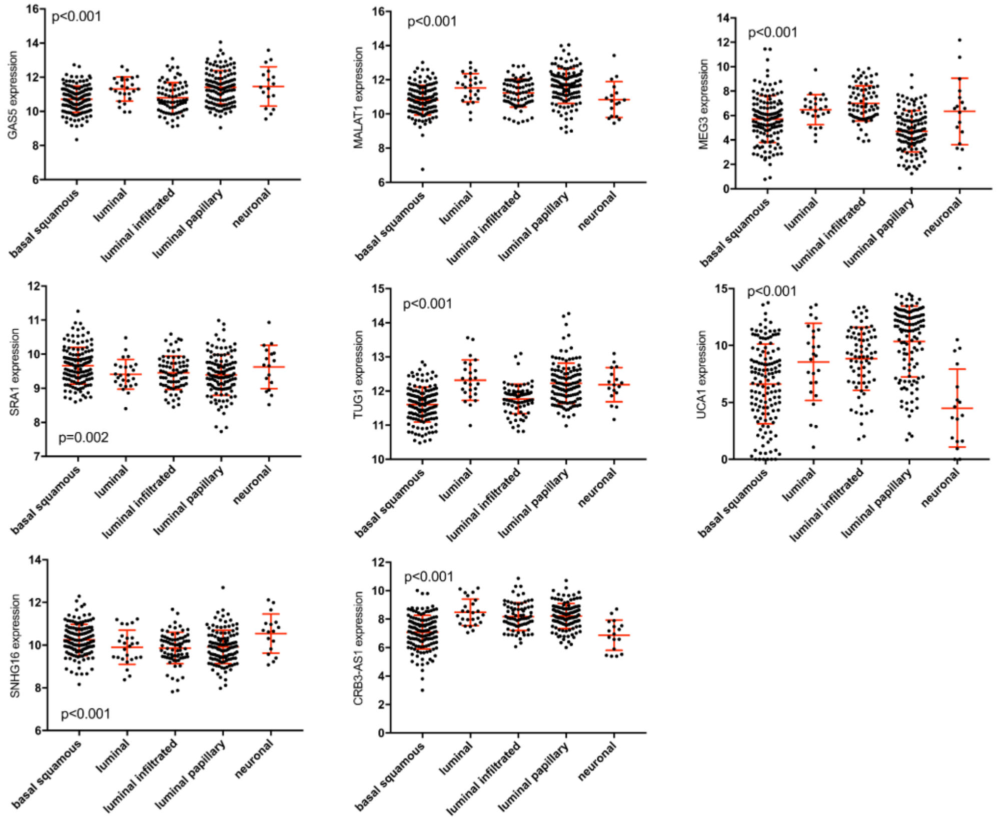

Figure S1: Patients of the TCGA cohort (n=371) clustered along the molecular MIBC subtypes as defined by the TCGA study. Differential lncRNA expression between the different molecular TCGA MIBC subtypes.

Figure S2: Mannheim cohort:

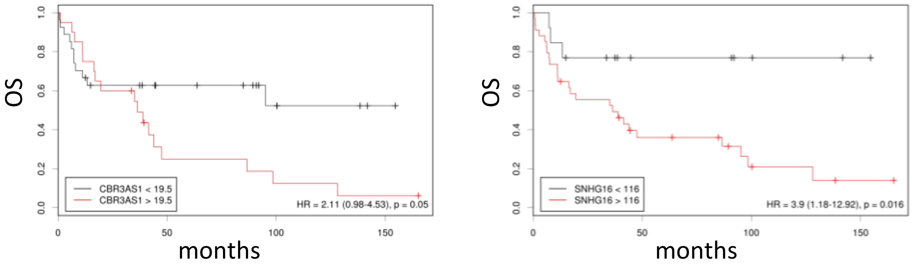

Figure S2: Kaplan Meier plots of LncRNAs allowing a risk stratification based on high and low transcript level expression in the Mannheim cohort. OS = Overall survival

Figure S3: TCGA cohort

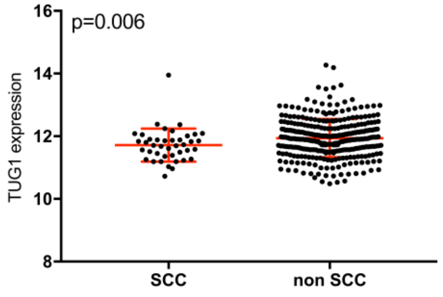

Figure S3: Differential expression of TUG1 in squamous and non-squamous MIBC of the TCGA cohort (n=371).
