# Supplementary material for: Subtype specific expression and survival prediction of pivotal lncRNAs in muscle invasive bladder cancer
Source: Sci Rep. 2020 Nov 24;10:20472. doi: 10.1038/s41598-020-77252-2 (PMC7687888; doi:10.1038/s41598-020-77252-2)
Supplement: Supplementary file 2 — Supplementary Table S1. [file 41598_2020_77252_MOESM2_ESM.docx]

**Nanostring OS**

| lncRNA | HR (high vs. Low) | 95% CI | P-value |
| --- | --- | --- | --- |
| BLACAT | 1.87 | 0.81-5.10 | 0.1500 |
| CBR3AS1 | 2.12 | 0.10-4.68 | 0.0507 |
| CTBP1AS | 2.17 | 0.89-4.87 | 0.0862 |
| GAS5 | 2.50 | 0.96-8.54 | 0.0604 |
| H19* | 1.57 | 0.69-3.38 | 0.2673 |
| HOTAIR | 1.55 | 0.57-3.63 | 0.3633 |
| MALAT1 | 2.52 | 0.98-6.09 | 0.0538 |
| MEG3 | 0.57 | 0.26-1.20 | 0.1382 |
| SNHG16 | 3.89 | 1.36-16.38 | **0.0084** |
| SRA1 | 0.66 | 0.29-1.69 | 0.3655 |
| TP53COR1 | 2.12 | 0.83-7.23 | 0.1291 |
| TUG1 | 1.56 | 0.72-3.29 | 0.2542 |
| UCA1 | 0.59 | 0.26-1.43 | 0.2264 |
| XIST | 1.50 | 0.71-3.32 | 0.2901 |

**Nanostring DSS**

| lncRNA | HR (high vs. Low) | 95% CI | P-value |
| --- | --- | --- | --- |
| BLACAT | 1.32 | 0.44-4.81 | 0.6364 |
| CBR3AS1** | 3.49 | 1.16-12.83 | **0.0259** |
| CTBP1AS | 1.51 | 0.34-4.99 | 0.5475 |
| GAS5 | 1.58 | 0.49-7.03 | 0.4641 |
| H19* | 4.04 | 1.38-12.48 | **0.0060** |
| HOTAIR | 0 | 0-infinite | **0.0366** |
| MALAT1 | 2.74 | 0.69-9.78 | 0.1400 |
| MEG3 | 0.57 | 0.19-1.63 | 0.2914 |
| SNHG16 | 2.88 | 0.78-18.56 | 0.1192 |
| SNHG16* | 3.06 | 1.02-11.20 | **0.0480** |
| SRA1 | 0.36 | 0.12-1.19 | 0.0887 |
| TP53COR1 | 1.33 | 0.41-5.89 | 0.6538 |
| TUG1 | 1.62 | 0.53-4.67 | 0.3838 |
| UCA1 | 0.40 | 0.14-1.33 | 0.1277 |
| XIST | 1.01 | 0.35-2.98 | 0.9785 |

**Chungbuk OS**

| lncRNA | HR (high vs. Low) | 95% CI | P-value |
| --- | --- | --- | --- |
| GAS5 | 3.06 | 1.20-10.32 | **0.0163** |
| H19 | 2.43 | 1.07-5.08 | **0.0354** |
| MALAT1 | 0.47 | 0.23-0.98 | **0.0429** |
| MEG3 | 2.42 | 1.10-6.09 | **0.0263** |
| SRA1 | 0.74 | 0.33-1.99 | 0.0523 |
| TUG1 | 1.43 | 0.71-2.99 | 0.3201 |
| XIST | 2.85 | 1.42-5.70 | **0.0037** |

**Chungbuk DSS**

| lncRNA | HR (high vs. Low) | 95% CI | P-value |
| --- | --- | --- | --- |
| GAS5 | 2.51 | 0.97-8.54 | 0.0588 |
| H19 | 2.57 | 1.13-5.86 | **0.0200** |
| MALAT1 | 0.46 | 0.21-1.02 | **0.0440** |
| MEG3 | 3.93 | 1.35-11.39 | **0.0068** |
| SRA1 | 0.77 | 0.32-1.29 | 0.6041 |
| TUG1 | 1.4 | 0.68-3.27 | 0.3405 |
| XIST | 2.96 | 1.35-6.47 | **0.0044** |

**MDA OS**

| lncRNA | HR (high vs. Low) | 95% CI | P-value |
| --- | --- | --- | --- |
| MEG3 | 0.78 | 0.30-1.78 | 0.5726 |
| TUG1 | 0.97 | 0.41-2.14 | 0.9451 |
| SRA1 | 0.49 | 0.20-1.34 | 0.1528 |
| MALAT1 | 0.47 | 0.11-1.36 | 0.1782 |
| H19 | 0.37 | 0.11-0.99 | **0.0465** |
| UCA1 | 0.39 | 0.18-0.88 | **0.0242** |
| HOTAIR | 1.45 | 0.67-3.26 | 0.3449 |

**MDA DSS**

| lncRNA | HR (high vs. Low) | 95% CI | P-value |
| --- | --- | --- | --- |
| MEG3 | 1.44 | 0.52-3.75 | 0.4681 |
| TUG1 | 0.25 | 0.06-1.09 | **0.0460** |
| SRA1 | 0.45 | 0.16-1.59 | 0.1933 |
| MALAT1 | 0 | 0-inf | **0.0310** |
| H19 | 0.29 | 0.04-1.04 | 0.0590 |
| UCA1 | 0.52 | 0.20-1.52 | 0.2198 |
| HOTAIR | 2.01 | 0.76-5.84 | 0.1611 |

**TCGA OS**

| lncRNA | HR (high vs. Low) | 95% CI | P-value |
| --- | --- | --- | --- |
| GAS5 | 0.58 | 0.34-1.08 | 0.0822 |
| H19 | 1.41*10^9^ | 3.26-3.26 | **<0.001** |
| MALAT1 | 0.51 | 0.36-0.75 | **<0.001** |
| MEG3 | 1.63 | 0.98-2.96 | 0.0616 |
| SRA1 | 1.31 | 0.84-1.95 | 0.2286 |
| TUG1 | 0.64 | 0.46-0.89 | **0.0244** |
| UCA1 | 0.33 | 0.18-0.71 | **0.0063** |
| SNHG16 | 1.43 | 1.00-2.03 | **0.0460** |
| CBR3-AS1 | 0.63 | 0.45-0.87 | **0.0046** |
| XIST | n.a. | n.a. | n.a. |
| HOTAIR | n.a. | n.a. | n.a. |

**Table S1:** Cohort summaries of the 95% confidence intervals (95%CI) and hazard ratios calculated by an univariable proportional-hazards model.
